# The Case of Kitson vs. Playfair

**Published:** 1896-05

**Authors:** 


### THE CASE OF KITSON VS. PLAYFAIR.

This late case, which, from the prominence of the parties and the nature of the charges, has been of such interest in London and elsewhere, will probably create a precedent among cases of its class in determining how far physicians may be justified in communicating professional secrets to their families.

Mrs. Kitson being ill, sent for Dr. Playfair, who, by the way, was the husband of her husband's sister. Mrs. Kitson's husband had been absent from London for more than a year. From examination of the plaintiff Dr. Playfair was convinced that she had not been a virtuous wife, and the doctor so told members of his own family in order to guard them against a woman who, in his opinion, was unfit for their society. Mrs. Playfair's brother (Sir Jas. Kitson) them withdrew an annuity of $2,500 which he had been accustomed to allow the Kitsons.

Hence a suit against Dr. Playfair for $60,000 damages. The defendant, instead of pleading the truth of his statements, set up as a defense that his communication of the alleged facts was based upon his honest belief and was privileged. Neither the judge nor the jury, however, took this view of it, and both were said to be in sympathy with the plaintiff. Mrs. Kitson was given a verdict for the full amount of damages, and the judge refused to allow the case to be appealed.

This is certainly one case where silence would have been golden.

---

WE are requested by Dr. C. M. Drake, the Chief Surgeon of the Southern Railway Company, to announce to members of the American Medical Association, that the Southern Railway Company will give to the members and their families a complimentary excursion on Wednesday, May 6, 1896, from Atlanta to Lithia Springs, Ga., and return, on the occasion of an old-fashioned "Georgia barbecue," to be given at the Springs on that day by Mr. Marsh, under the supervision of the Committee of Arrangements of the American Medical Association.

The Chief Surgeon of the Southern Railway Company has also the pleasure of announcing a complimentary journey to be extended to a limited number of distinguished members of the American Medical Association, to leave Atlanta on the evening of Friday, May 8, to visit Lookout Mountain and Tate Epsom Springs, Tennessee, Hot Springs, Asheville, and the "Land of the Sky," Western North Carolina, returning to Atlanta. Invitations for this excursion will be issued to members of the Association after registration with the permanent Secretary.

